# The mitochondrial genome of *Erannis ankeraria* (Lepidoptera: Geometridae)

**DOI:** 10.1080/23802359.2019.1644560

**Published:** 2019-07-22

**Authors:** Yimeng Chen, Qinghua Wang, Shaobo Wang, Liangjian Qu

**Affiliations:** a College of Plant Protection, Hainan University, Haikou, China;; b Research Institute of Forest Ecology, Environment and Protection, Chinese Academy of Forestry, Beijing, China;; c Academy of Forestry, Henan University of Science and Technology, Luoyang, China

**Keywords:** Mitochondrial genome, Ennominae, phylogenetics

## Abstract

The measuring worm *Erannis ankeraria* belongs to the subfamily Ennominae of Geometridae. The mitogenome (GenBank accession number: MN046105) of *E. ankeraria* was sequenced, the new representative of the mitogenome of the subfamily. The nearly complete mitogenome is 15,250 bp totally, consisting of 13 protein-coding genes, two rRNAs, and 22 transfer RNAs. All genes have the similar locations and strands with that of other published species of Geometridae. The nucleotide composition biases towards A and T, which together made up 79.3% of the entirety. Bayesian inference analysis strongly supported the monophyly of Bombycoidea, Geometroidea, Noctuoidea, Papilionoidea, Pyraloidea, and Tortricoidea were strongly supported. This result also suggested that the Geometroidea was the sister to Bombycoidea, and then Noctuoidea was assigned to the sister group to the clade of Geometroidea + Bombycoidea, and then Pyraloidea was the sister group to the clade that contains Geometroidea, Bombycoidea, and Noctuoidea, and then Papilionoidea was the sister group to the clade that contains these four superfamilies mentioned above.

## Introduction

Lepidoptera is one of the world largest insect orders, second only to Coleoptera, with approximately 157,424 described species (van Nieukerken et al. 2011). The controversial issues of the phylogenetic relationships in the Lepidoptera have been intensively discussed, at both deep-level and low-level (Fibiger et al. 2010; Mutanen et al. 2010; Zahiri et al. 2012).

The specimens of *E. ankeraria* used for this study were collected in Xinghe County of Inner Mongolia Autonomous region by Qinghua Wang and identified by Yimeng Chen. Specimens are deposited in the Research Institute of forest ecological environment and protection, Chinese Academy of Forestry with the deposit number: CAF NO.V20170607. The total genomic DNA was extracted from the whole body (except head) of the specimen using the QIAamp DNA Blood Mini Kit (Qiagen, Germany) and stored at −20 °C until needed. The mitogenome was sequenced in Allwegene technology limited company with the sequenced number: AWGT19042503. The nearly complete mitogenome of *E. ankeraria* is 15,250 bp (GenBank accession number: MN046105). It encoded 13 PCGs, 22 tRNA genes, two rRNA genes and were similar with related reports before (Hong et al. 2009; Hao et al. 2012; Chen et al. 2016; Zheng et al. 2018). All genes have the similar locations and strands with that of other published Geometridae species. The nucleotide composition of the mitogenome was biased toward A and T, with 79.3% of A + T content (A = 41.3%, T = 38.0%, C = 12.3%, and G = 8.4%). The A + T content of PCGs, tRNAs, and rRNAs is 77.7%, 81.5%, and 85.5%, respectively. The total length of all 13 PCGs of *E. ankeraria* is 11,293 bp. Two PCGs (*NAD2* and *NAD5*) initiated with ATT codons, and six PCGs (*COII*, *COIII*, *ATP6*, *NAD4*, *NAD4L*, and *CYTB*) initiated with ATG codons, *NAD1*, *NAD6*, and *NAD3* initiated with ATA as a start codon, start codon of *CO1* not determined. Twelve PCGs used the typical termination codons TAA except *NAD4* used T in *E. ankeraria.*

**Figure 1.**
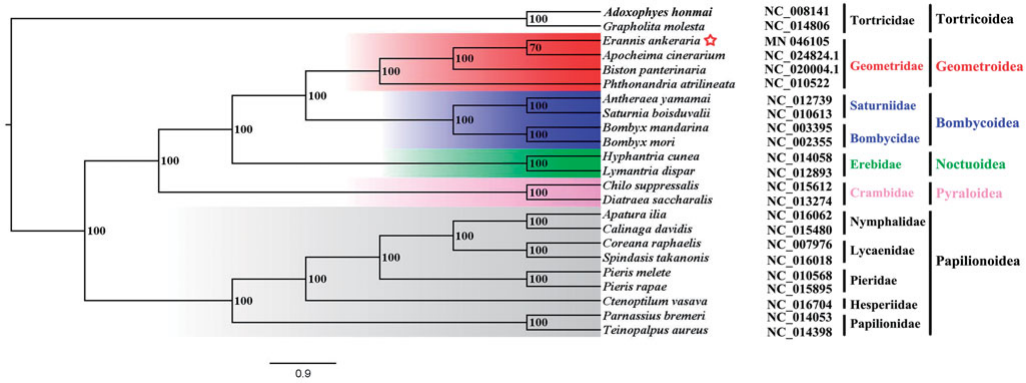
Bayesian phylogenetic tree of 23 Lepidoptera species. The posterior probabilities are labeled at each node. Genbank accession numbers of all sequence used in the phylogenetic tree have been included in the Figure 1 and corresponding to the species names.

Phylogenetic analysis was performed based on the nucleotide sequences of 13 PCGs from 23 Lepidoptera species. Bayesian (BI) analysis generated the phylogenetic tree topologies based on the PCGs matrices (Figure 1). According to the phylogenetic result, the monophyly of Bombycoidea, Geometroidea, Noctuoidea, Papilionoidea, Pyraloidea, and Tortricoidea were strongly supported. The Geometroidea was the sister to Bombycoidea, Noctuoidea was assigned to the sister group to the clade of Geometroidea + Bombycoidea, Pyraloidea was the sister group to the clade that contains Geometroidea, Bombycoidea, and Noctuoidea, and then Papilionoidea was the sister group to the clade that contains these four superfamilies mentioned above. This result that the phylogenetic relationship among five superfamilies mentioned above is consistent with the phylogenetic result of the previous study (Yang et al. 2013). The nearly complete mitogenome of *E. ankeraria* could provide the important information for the further studies of Lepidoptera phylogeny.
